# Dicer-2 mutations in Aedes aegypti cells lead to a diminished antiviral function against Rift Valley fever virus and Bunyamwera virus infection

**DOI:** 10.1099/jgv.0.002046

**Published:** 2024-11-07

**Authors:** Susann Dornbusch, Melinda Reuter, Rhys H. Parry, Michael Stern, Stefanie C. Becker, Esther Schnettler

**Affiliations:** 1 Institute for Parasitology and Research Center for Emerging Infections and Zoonoses, University of Veterinary Medicine Hannover, Hannover, Germany; 2 Institute for Physiology and Cell Biology, University of Veterinary Medicine Hannover, Hannover, Germany; 3 Bernhard-Nocht-Institute for Tropical Medicine, Hamburg, Germany; 4 School of Chemistry and Molecular Biosciences, The University of Queensland, St. Lucia, Australia; 5 Faculty of Mathematics, Informatics and Natural Sciences, University of Hamburg, Hamburg, Germany; 6 German Center for Infection Research (DZIF), partner side HH-HL-Bo-Ri, Hamburg, Germany

**Keywords:** antiviral RNAi, arbovirus, Bunyaviricetes, Dcr2, mosquito, RVFV

## Abstract

Mosquitoes are known to transmit different arthropod-borne viruses belonging to various virus families. The exogenous small interfering RNA pathway plays an important role in the mosquito defence against such virus infections, with Dicer-2 (Dcr2) as one of the key proteins that initiates the cleavage of viral dsRNAs into 21 nt long virus-derived small interfering RNAs. Previous data identified the importance of various motifs in Dcr2 for its small interfering RNA (siRNA)-mediated antiviral activity. However, all these data focus on positive-strand RNA viruses, although negative-strand RNA viruses, like *Bunyaviricetes*, include several important mosquito-borne viruses. Here, we aim to investigate the importance of different domains of Dcr2 for antiviral activity against viruses of the *Bunyaviricetes*. For this, we used the *Aedes aegypti-*derived Dcr2 knock-out cell line Aag2-AF319 to study the importance of the helicase, RNase III and PIWI–Argonaute–Zwille domains of Dcr2 on the antiviral activity of two viruses belonging to different families of the *Bunyaviricetes*: the Rift Valley fever virus (RVFV) vaccine strain MP12 (*Phenuiviridae*, *Phlebovirus*) and the Bunyamwera orthobunyavirus (BUNV; *Peribunyaviridae*, *Orthobunyavirus*). All three domains were determined to be critical for the antiviral activity against both RVFV and BUNV. Interestingly, one specific mutation in the helicase domain (KN) did not result in a loss of antiviral activity for RVFV, but for BUNV, despite losing the ability to produce 21 nt siRNAs.

## Introduction

As the world currently faces an emerging threat of arthropod-borne virus infections, it becomes increasingly important to understand and assess every part of the arboviral life cycle, transmission and antiviral responses in the vertebrate host and invertebrate vector. Mosquitoes transmit various arboviruses belonging to different virus families, including dengue virus (DENV; *Flaviviridae*, *Orthoflavivirus*), chikungunya virus (CHIKV; *Togaviridae*, *Alphavirus*) and Rift Valley fever virus (RVFV; *Phenuiviridae*, *Phlebovirus*), to name but a few [1,8].

Mosquitoes actively fight arbovirus infection using various immune responses. These include the RNA interference (RNAi) response with the exogenous small interfering RNA (exo-siRNA) pathway known as a major antiviral response in mosquitoes. This pathway is initiated by the recognition of long viral dsRNA molecules as a pathogen-associated molecular pattern by the RNase III endoribonuclease: Dicer-2 (Dcr2) [9,10]. Subsequently, these dsRNAs are cleaved by Dcr2, into 21 nt long viral small interfering RNAs (vsiRNAs) [11]. The vsiRNAs are loaded onto Argonaute-2 (Ago2), which is part of the small interfering RNA (siRNA)-induced silencing complex. Ago2 unwinds the vsiRNAs and retains one strand for recognition and subsequent degradation and silencing of complementary viral RNAs (vRNAs) [12].

The importance of Dcr2 has been illustrated by Dcr2 knock-out (KO) *Aedes aegypti* mosquitoes and Dcr2 KO *Ae. aegypti*-derived cell lines. In Dcr2 KO mosquitoes, some arbovirus infections (DENV, CHIKV, Zika virus and Mayaro virus) resulted in exacerbated viral replication in the early stages of infection and heightened the systemic viral dissemination, while the vector competence of the mutant mosquitoes was found to be comparable to WT mosquitoes [13]. In other studies, Dcr2 KO *Ae. aegypti* mosquitoes showed a higher mortality to Sindbis virus and Yellow fever virus infection [14]. Along these lines, increased virus replication was observed for various arboviruses of different families in *Ae. aegypti*-derived Dcr2 KO cells, including members of the *Bunyaviricetes* [15,18]. These results highlight the importance of the Dcr2 protein in the antiviral response in mosquitoes as well as its effect on vector competence and capacity. However, most knowledge about the interaction of the RNAi response and arboviruses has been investigated for positive-strand RNA viruses, whereas relatively little is known about RNAi in the context of infections caused by negative single-stranded (-ss) RNA viruses. As negative-strand RNA viruses produce lower levels of dsRNA during infection in comparison to positive-strand RNA viruses [19,20], less or even no recognition by Dcr2 and the corresponding antiviral activity of the exo-siRNA pathway could be expected. Sequence analysis of Dcr2 in *Ae. aegypti* identified several domains, which are mostly conserved between Dcr2 of different organisms, supporting their importance. Within this study, the focus lies on several of these domains, namely, the helicase domain, with its main function being the binding of blunt-end dsRNA [21,24], as well as the PIWI–Argonaute–Zwille (PAZ) and the two RNase III domains, RNase IIIA and RNase IIIB, which together are considered to be the functional core of Dcr2. The PAZ domain is responsible for binding of 3′ overhanging dsRNA [25], while the RNase III domains are required to process the dsRNA into typically 21 nt siRNAs and binding to Ago2 [26,27].

Until now, only a handful of experiments have been performed to investigate the above-mentioned, hypothesized function of the different domains of *Ae. aegypti* Dcr2 or determine their importance for the antiviral activity of Dcr2. Mutational analysis, followed by infection with Semliki Forest alphavirus (SFV; a positive-strand RNA virus), has shown that the helicase, RNase III and PAZ domains are important for the siRNA-based antiviral activity of Dcr2 in *Ae. aegypti*-derived cells as well as the vsiRNA production [28,29]. For this, multiple sequence alignment was used to identify highly conserved aas within these domains, leading to the production of multiple different Dcr2 mutants, some of which were used within this study, namely, two helicase mutants, K39N (abbreviated to KN) and G488R (abbreviated to GR); the RNase III A and B mutant mtR3; and the PAZ mutants M1 and M3. After transfection of WT or mutant Dcr2 and SFV infection, all mentioned mutants exhibited a significant decrease in antiviral activity against SFV, as well as an impaired 21 nt SFV-specific siRNA production [28,29].

Arboviruses of the order *Elliovirales* and *Hareavirales*, both belonging to the *Bunyaviricetes*, account for a high proportion of medically and livestock-important mosquito-borne viruses. However, there is only limited knowledge about their interaction with the antiviral RNAi response in mosquitoes and the importance of the different Dcr2 domains regarding their antiviral activity against representative viruses of the *Bunyaviricetes*, such as RVFV and Bunyamwera orthobunyavirus (BUNV; *Peribunyaviridae*, *Orthobunyavirus*) [18,30,32]. This includes reports of orthobunya- and phlebovirus-specific small RNAs in infected mosquitoes and derived cell lines. In addition, the antiviral activity of some key RNAi proteins, e.g. Dcr2, for a small selection of *Bunyaviricetes* was shown [18].

RVFV and BUNV both share a similar genomic organization, consisting of a tri-segmented (-ss) RNA genome with different segment sizes. The L segment (RVFV: 6.4 kb; BUNV: 6.9 kb) encodes for the RNA-dependent RNA polymerase needed for the replication and transcription of the virus genome [33,34]. The M segment (RVFV: 3.8 kb; BUNV: 4.4 kb) contains sequences for the structural glycoproteins Gn and Gc, enabling virus particle entry into host cells and, in the case of RVFV, sequences for a non-structural 78 kDa accessory protein and the NSm protein, which plays an important role for RVFV replication and dissemination within the mosquito [35]. The coding strategy of the S segment (RVFV: 1.7 kb; BUNV: 0.9 kb) differs between BUNV and RVFV, with the latter using an ambisense coding strategy via a hairpin structure instead of a negative-strand coding strategy as in BUNV. For both viruses, the S segment encodes for a non-structural NSs protein (known to counteract the host immune responses) [36,37] and a nucleocapsid protein (N). The latter is responsible for the encapsidation of vRNA genome into ribonucleoprotein complexes [38,45].

**Fig. 1.**
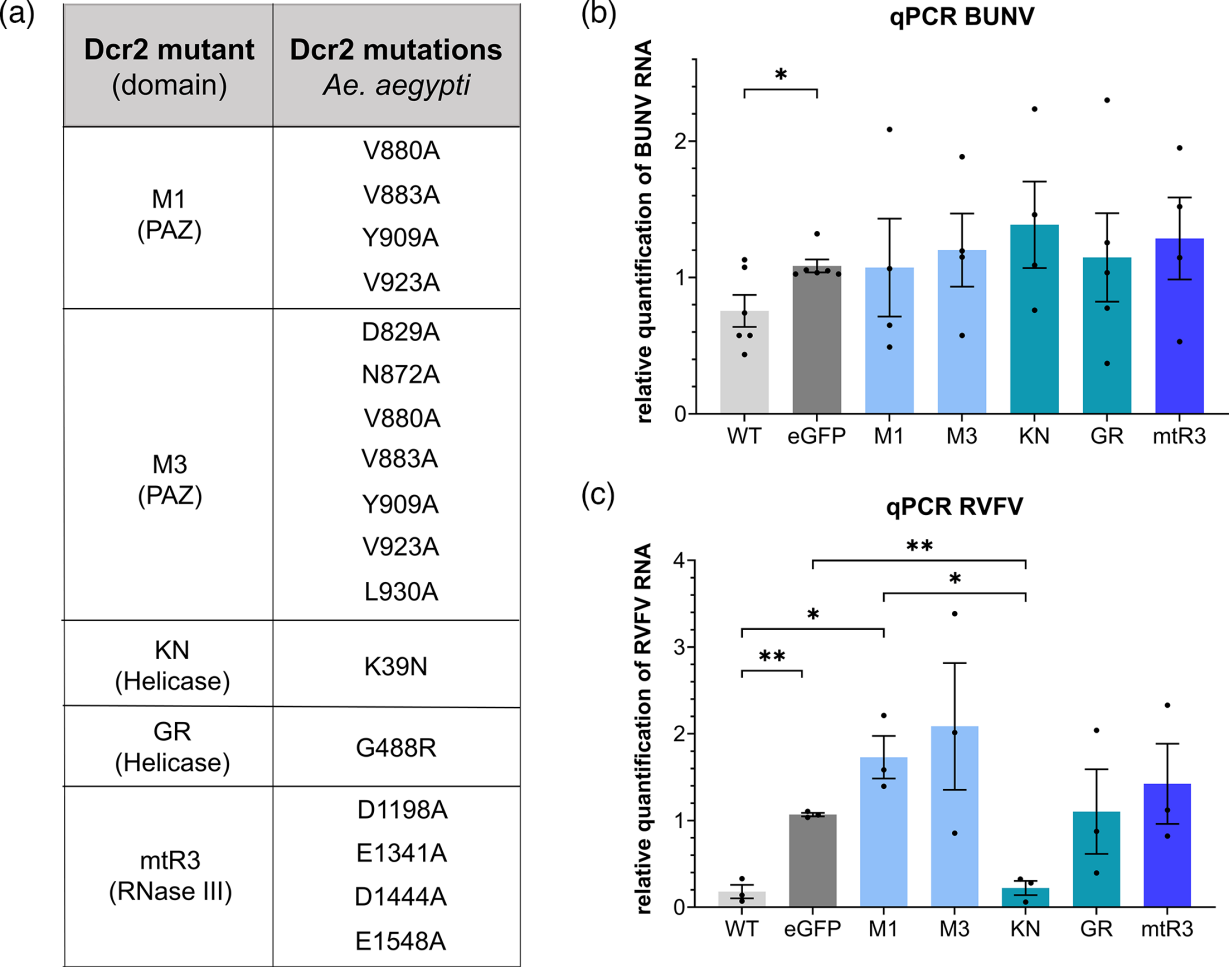
Antiviral activity of different mutant *Ae. aegypti* Dcr2 against RVFV and BUNV. (**a**) Table of Dcr2 mutants with corresponding mutations and their domain location. (**b**) Effect of mutant Dcr2 on BUNV and (**c**) RVFV vRNA. Aag2-AF319 cells were transfected with pPUb-plasmids expressing WT or mutant Dcr2s, with pPUb-myc-EGFP as control. The cells were infected at 24-h post-transfection (hpt) with BUNV or RVFV (both MOI 0.1), respectively. At 72-h post-infection (hpi), total RNA was isolated and analysed using qRT-PCR. Relative quantification (RQ) was calculated using S7 rRNA as a housekeeper gene for qRT-PCR and EGFP as control. The results are shown as sem of at least three independent biological replicates. The dots represent different biological replicates, consisting of two technical replicates each with * = *P* < 0.05 and ** = *P* < 0.01 according to Student’s *t*-test.

In this study, Dcr2-deficient *Ae. aegypti*-derived cells were used to investigate the antiviral activity of previously identified Dcr2 mutants against RVFV MP12 (abbreviated to RVFV) and BUNV (Fig. 1a). Except for the helicase mutant, KN, after RVFV infection, all mutants exhibited an antiviral activity against both RVFV and BUNV similar to the EGFP-transfected negative control. Surprisingly, small RNA sequencing supported the inability of KN to produce 21 nt RVFV-specific small RNAs, similar to GR. These findings are important for further understanding the effect of Dcr2 and its domains on negative-strand RNA viruses.

## Methods

### Cells

In this study, we used the Aag2-AF319 (ECACC 19022602; Public Health England) cells, which contain a Dcr2 KO mutation and were derived from Aag2-AF5 (ECACC 19022601, Public Health England) embryonic cells [46]. For Aag2-AF319 cell maintenance, Leibovitz’s L-15 medium (Gibco Thermo Fisher Scientific Inc., Waltham, MA, USA) was supplemented with 10% FBS (Gibco Capricorn Scientific GmbH, Ebsdorfergrund, Germany), 10% tryptose phosphate broth (Gibco Thermo Fisher Scientific Inc.) and penicillin-streptomycin (100 U ml^−1^-100 µg ml^−1^; PAN-Biotech, Aidenbach, Germany). The cells were grown at 28 °C without CO_2_ and passaged twice a week.

### Viruses

BUNV (accession numbers: NC_001927.1, NC_001926.1 and NC_001925.1) was produced in BHK-21 cells with an MOI below 1, and the titre was determined by plaque assays as previously described for Schmallenberg virus [47]. RVFV MP12 (abbreviated to RVFV; provided by Professor Elliot, CVR, UK) was amplified in BHK-21 cells with an MOI below 1. On day 3, the virus was harvested via polyethylene glycol precipitation, and the titre was determined via the TCID_50_ method. The viruses were stored at −80 °C.

### Plasmids

For Aag2-AF319 KO cell line transfection experiments, previously described expression plasmids pPUb-myc-Dcr2 WT and pPUb-myc-EGFP or pPUb-plasmids expressing mutant Dcr2 (KN, GR, M1, M3 and mtR3) were used [28,29]. While the two helicase mutants KN (K39N) and GR (G488R) possess one eponymous mutation each, the RNase III mutant mtR3 (D1198A, E1341A, D1444A and E1548A) and the two PAZ mutants M1 (V880A, V883A, Y909A and V923A) and M3 (D829A, N872A, V880A, V883A, Y909A, V923A and L930A) have multiple different mutations.

### Assessment of antiviral activity of mutant Dcr2 via qPCR and statistical analysis

For determination of the antiviral activity of the mutant Dcr2 against BUNV and RVFV, 2×10^5^ Aag2-AF319 cells/well were seeded in 24-well plates. After the cells were allowed to settle for at least 4 h, the cells were transfected with 500 ng expression plasmids: WT Dcr2, mutant Dcr2s or EGFP (as control) and 1 µl DharmaFECT 2 (Horizon Discovery Ltd, Cambridge, UK). At 24-h post-transfection (hpt), the cells were infected with either BUNV or RVFV (MOI 0.1) for 2 h at 28 °C, followed by replacement with fresh complete media. For total RNA extraction, the cells were lysed using TRIzol (Thermo Fisher Scientific Inc.; BUNV) or QIAzol (Qiagen, Hilden, Germany; RVFV) at 72-h post-infection (hpi), according to the manufacturer’s protocol. One microlitre of total RNA was used to detect the RVFV L segment or S7 rRNA (as housekeeper) via Luna Universal Probe One-Step RT-qPCR Kit from New England Biolabs GmbH (Frankfurt am Main, Germany) according to the manufacturer’s instructions via the AriaMx Real-Time PCR system (Agilent Technologies, Santa Clara, CA, USA). The previously described primers [30] were used. For BUNV detection, 1.5 µg total RNA was first reverse transcribed with MML-V (Promega Corporation, Madison, WI, USA) and random hexamers, according to the manufacturer’s protocol. To quantify BUNV and S7 rRNA (as housekeeper), 1 µl cDNA was used together with the QuantiTect SYBR Green PCR Kit (Qiagen) and the LightCycler 480 System (Roche Diagnostics, Mannheim, Germany). For primers, see Table S1 (available in the online version of this article). To ensure effective control of Dcr2 protein expression, Western blot analysis was conducted with the results presented in Fig. S1. For statistical analysis of the RT-qPCR RVFV results, the Agilent Aria software v1.71 was used, while LightCycler 480 was used for two-step qPCR results of BUNV. The fold gene expression [2^-(∆∆Ct); relative quantification] was calculated using S7 rRNA as housekeeper and EGFP-transfected cells as control. The results were plotted and statistically tested using Student’s *t*-test via GraphPad Prism 10 (GraphPad Software, Boston, MA, USA).

### Small RNA sequencing and analysis

For small RNA sequencing, 2.5×10^5^ Aag2-AF319 cells/well were seeded in four wells of a 24-well plate and transfected with either 500 ng pPUb-myc-Dcr2 WT, pPUb-myc-Dcr2 KN, pPUb-myc-Dcr2 GR or pPUb-myc-EGFP (control) together with 1 µl DharmaFECT 2 (Horizon Discovery Ltd), followed by infection with RVFV MOI 1, at 24 hpt. For total RNA isolation at 48 hpi, four wells per construct were combined using 1 ml TRIzol (Thermo Fisher Scientific Inc.) and glycogen as a carrier, following the manufacturer’s protocol. Small RNA sequencing was performed at BMKGene (Münster, Germany) on an Illumina NovaSeq SP (Illumina, San Diego, CA, USA) for library preparation with SE50 and 10 M clean reads/sample. A minimum of 1.5 µg total RNA was sent per sample to BMKGene for the library preparation with the VAHTS Small RNA Library Prep Kit for Illumina (Vazyme Biotech, Nanjing, China) according to the manufacturer’s protocol. Small RNA sequencing data can be accessed at the NCBI Sequence Read Archive under BioProject ID: PRJNA1120211. Basecalled fastq files were quality and adapter trimmed using the fastp tool (v0.23.2) [48], retaining 16–30 nt reads under default conditions. The clean, trimmed reads were then mapped to the RVFV genome (GenBank ID: L segment DQ375404.1, M segment DQ380208.1 and S segment DQ380154.1) using Bowtie2 (v2.4.5) [49] with the sensitive mapping flag (–sensitive). Trimming and mapping statistic summary is available in Table S2. The histogram of mapped read lengths and first bp bias was generated using viral_sRNA_tools/3_bam_sRNA_histogram.sh script, which utilizes SAMtools (v1.16.1). Overlapping pair probabilities (*z*-score) of 25–30 nt reads were calculated using the small RNA signatures Python script signature.py [50], with the following conditions (–minquery 25 –maxquery 31 –mintarget 25 –maxtarget 30 –minscope 1 –maxscope 20). Biases in extracted PIWI-interacting RNAs (piRNAs) for both the genome and antigenome strands were visualized using WebLogo3 [51]. Output BAM files from Bowtie2 were filtered to include only the 21 nt reads for the vsiRNA mapping. Coverage statistics for each position of the RVFV genome were extracted using bedtools genome coverage tool (v2.27.1) [52] and visualized using GraphPad Prism (v10.0.2).

## Results

### Analysis of the antiviral activity of mutant Dcr2 against RVFV and BUNV

To investigate the importance of the different Dcr2 domains (helicase, PAZ and RNase III) for the antiviral activity against BUNV and RVFV, the Dcr2 KO cell line (Aag2-AF319) was used together with corresponding expression constructs. The cells were transfected with expression constructs for WT Dcr2, mutant Dcr2s (KN, GR, M1, M3 and mtR3; Fig. 1a) or EGFP (negative control), followed by infection with either BUNV or RVFV at MOI 0.1. The effect of the different constructs on BUNV (Fig. 1b) and RVFV (Fig. 1c) infection was determined by qRT-PCR. WT and mutant Dcr2 expression in AF319 Dcr2 KO cells was confirmed via Western blot (Fig. S1).

The cells transfected with WT Dcr2 showed reduced vRNA for both BUNV (*P* = 0.0266) and RVFV (*P* = 0.0051) (for *P*-values, see Table S3), compared to control cells (EGFP). In contrast, the transfection of most of the Dcr2 mutants (KN, GR, M1, M3 and mtR3) did not result in a statistically significant difference from the EGFP control. Surprisingly, RVFV RNA was significantly reduced in the cells transfected with the KN helicase mutant, compared to the EGFP-transfected negative controls (*P* = 0.0066). KN exhibited a ~79% decrease in vRNA, whereas GR, the second helicase mutant, showed similar levels of RVFV RNA than the EGFP control. Besides, for BUNV, no vRNA reduction was observed in the cells transfected with the KN mutant. With the exception of KN for RVFV, all other mutants show RVFV RNA levels comparable to the EGFP-transfected control. The quantification of infectious virus particles in the supernatant of the transfected and infected cells showed comparable results than observed for the vRNA (Fig. S2).

### Production of RVFV-specific 21 nt vsiRNAs in KN-transfected Dcr2 KO cells

It has been previously reported that in the case of SFV (*Togaviridae*, *Alphavirus*) infection, the mutants lost their ability to either produce vsiRNAs at all (for helicase and RNase III mutants) or the size of vsiRNAs was broadened from 21 nt to 18–23 nt in the case of the PAZ domain mutants. This depletion or broadening in length of produced vsiRNAs by the different mutants (KN, GR, M1, M3 and mtR3) leads to a loss or at least significant decrease in their antiviral activity against SFV as well as in dsRNA-based reporter assays. As the KN helicase mutant is still able to significantly reduce the RVFV infection, it was hypothesized that KN might be able to generate RVFV-specific 21 nt siRNAs in contrast to the ‘non-functional’ GR mutant. Therefore, small RNA sequencing was performed from Aag2-AF319 cells transfected with Dcr2 (KN and GR) expression constructs, followed by RVFV infection (MOI 1). As positive or negative control, WT Dcr2 or EGFP expression constructs were included. Total RNA was isolated at 48 hpi and small RNA sequencing was performed. The size distribution of all sequenced small RNA reads indicated a similar distribution of small RNA reads corresponding to siRNAs or micro RNAs (miRNAs) (21–22 nt) and piRNAs (24–30 nt) for all treatments (Fig. S3).

**Fig. 2.**
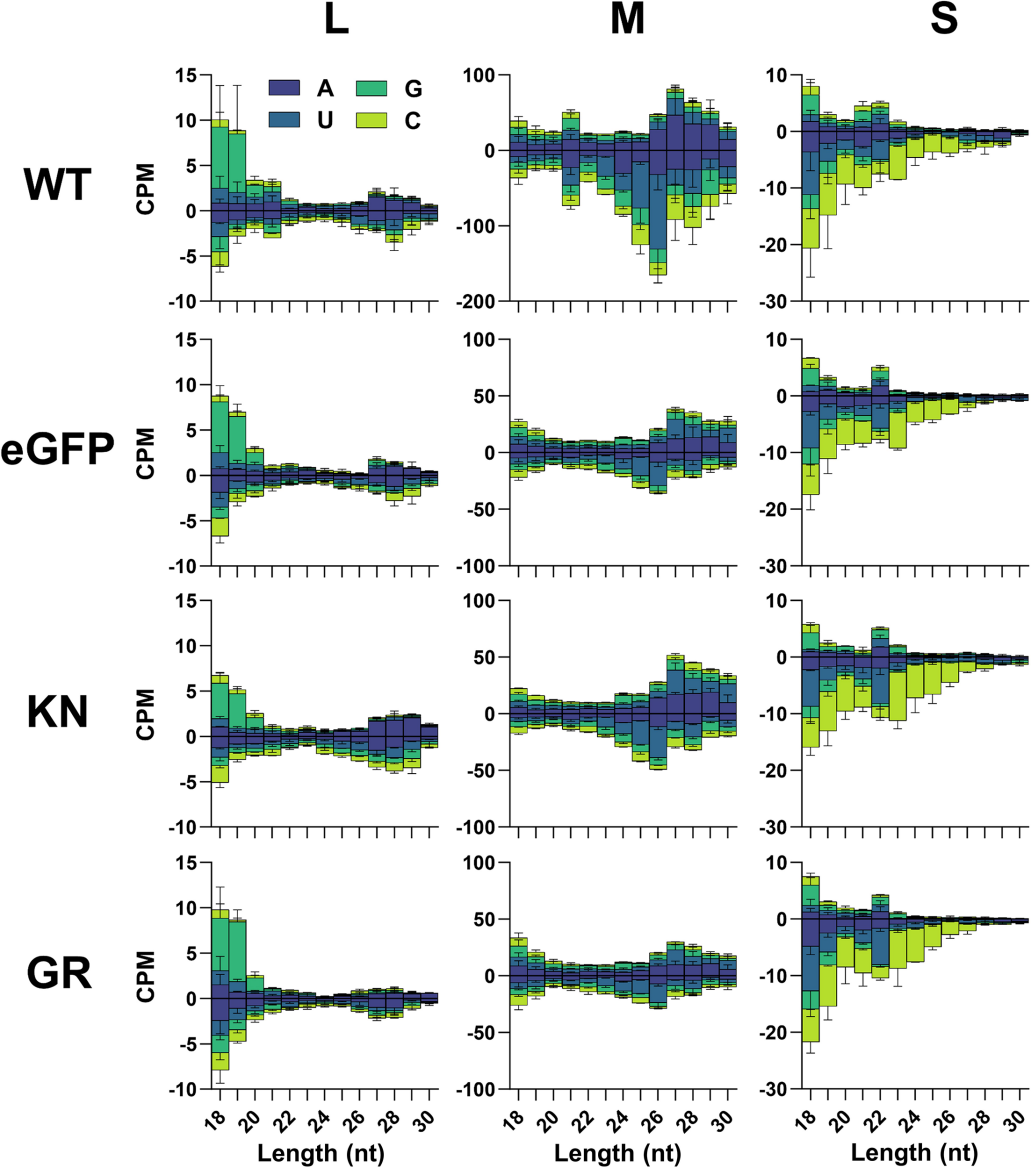
Mutations in Dcr2 in Aag-AF319 cells reduce the vsiRNA production against RVFV M segment. Small RNA sequencing of Aag2-AF319 cells transiently expressing WT Dcr2, EGFP, Dcr2 KN and Dcr2 GR mutations and infected with RVFV (MOI 1) at 48 hpi. Histogram of small RNA reads 18–30 nt in length, mapped to the RVFV antigenome (positive) and genome (negative) with colours indicating first base nt prevalence for each read length, shown as counts per million (CPM) from two independent experiments ± sd.

**Fig. 3.**
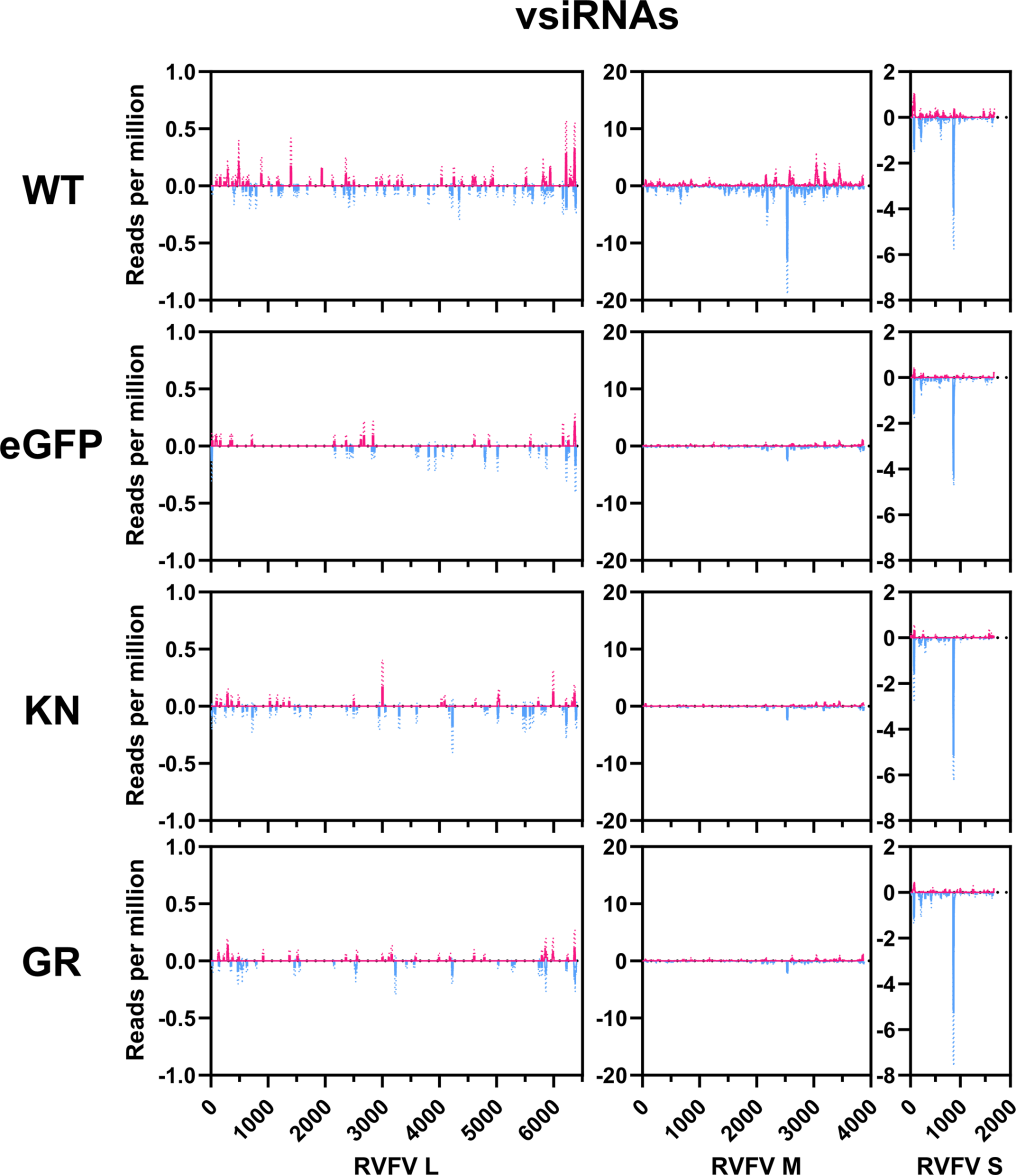
Differences in the mapping of 21 nt vsiRNAs in KN and GR Dcr2 mutants in RVFV infection. Coverage of RVFV-derived 21 nt vsiRNAs over the antigenome (magenta) or genome (cyan) (Y-axis, vsiRNA reads per million) for the L, M and S segments of RVFV from two independent experiments ± sd.

Bioinformatic analysis of small RNA libraries revealed the presence of viral-derived small RNA mapping to all three segments of the RVFV genome and antigenome for all treatments (Fig. 2). Overall, a greater number of small RNA mapping to the M segment than the S and L segments were detected (Table S2) for all conditions. Only the WT Dcr2 treatment showed an induction of vsiRNAs of 21 nt, again with amounts changing from M>S>L segment with the EGFP, KN and GR mutants lacking a 21 nt peak for any genome segment of RVFV associated with vsiRNAs. For the cells expressing WT Dcr2, the 21 nt vsiRNAs map along the whole M and the L segments from the genome and antigenome. Interestingly, in the case of the S segment, most 21 nt vsiRNAs map to 1 region (~900 nt) at the genome, corresponding with the stem-loop sequence in the S segment of RVFV (Fig. 3).

**Fig. 4.**
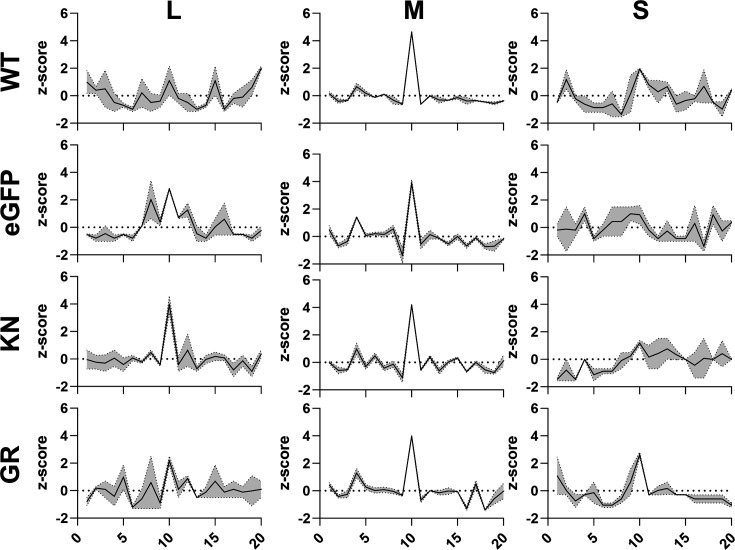
Limited differences in vpiRNA generation of Aag2-AF319 cells with Dcr2 WT, KN and GR mutants and EGFP control during RVFV infection. Probability of overlapping pairs *z*-scores of 25–30 nt virus-derived small RNAs. Mean of *n*=2 independent repeats is presented with the grey region indicating the range.

For cells expressing the EGFP or the helicase mutants, a high amount of RNAs with a length of 22 nt was found for the S segment (Fig. 2). Such vsRNAs were also observed in the cells with WT Dcr2, however, to a lower amount. None of the RNAs between 20 and 23 nt showed a bias in their first base. In WT-transfected cells, in addition to the 21 nt vsiRNAs, longer small RNAs were detected in the length of 24–30 nt, especially for the M segment. Interestingly, a reduction of these RNAs can be observed in EGFP, KN and GR mutants. Small RNAs with a length of 25 and 26 nt mapping to the M segment in the WT Dcr2-expressing cells mostly are derived from the genomic strand (Figs 2 and S4). In the cells expressing WT Dcr2, the 25–30 nt sRNA mapping to the L and M segments shows the ping-pong-produced viral piRNA (vpiRNA) characteristics: overlap of 10 nts between sense and antisense small RNAs and U_1_ and A_10_ bias. For all other treatments, an enriched *z*-score in overlapping sense and antisense small RNAs of 10 nts (Fig. 4) for the M segment as well as a U_1_ bias for the genomic sRNAs for the L and M segments is present (Fig. S5). However, in all treatments, there was insufficient vpiRNAs generated against the S segment, to observe these biases.

Mapping of these 25–30 nt ‘vpiRNAs’ along the genome and antigenome revealed no difference between the treatments, with the L segment targeted mostly at the 5′ and 3′ ends and the S segment derived 25–30 nt mapping mostly to 1 region (~900 nt) at the genome, as already observed for the 21 nt vsiRNAs (Fig. S4).

## Discussion

Dcr2 is known as key protein in the antiviral exo-siRNA pathway in *Ae. aegypti* for various viruses. It binds long dsRNA, cleaves it into 21 nt long siRNAs and loads them onto another key protein: Ago2. To perform these different tasks, Dcr2 has various domains, including the helicase, PAZ and RNase III domains. Previous experiments have shown the importance of these domains for the antiviral activity against SFV (a positive-strand RNA virus) and linked it to the ability of Dcr2 to produce specifically 21 nt long vsiRNA molecules [28,29]. As positive-strand RNA viruses produce higher levels of dsRNA during replication than negative-strand RNA viruses like BUNV and RVFV, dsRNA-triggered RNAi might play a lesser role in their antiviral defence [20].

Aag2-AF319 (Dcr2 KO) cells transfected with Dcr2 helicase (GR and KN), PAZ (M1 and M3) and RNase III (mtR3) mutants were unable to decrease the BUNV infection in contrast to WT Dcr2, supporting a loss in antiviral activity of these mutants. Although similar results were observed for RVFV for most tested mutants, the helicase KN mutant still significantly reduced RVFV infection, comparable to WT Dcr2. The used RVFV MP12 vaccine strain is known to be attenuated in mammals; however, no attenuation has been reported in mosquitoes so far [53]. Therefore, it is expected that the observed results, especially for the KN mutant, would be the same with other RVFV strains, but additional experiments are needed to verify this. With the exception of the helicase KN mutant in combination with RVFV, these results are in line with previously published data of SFV [28]. This leads to the conclusion that all three domains are critical for the overall antiviral defence against positive- and negative-strand RNA arboviruses. The lower effect of WT Dcr2 on BUNV RNA and infectious virus particles, compared to RVFV, suggests a lower sensitivity of BUNV to the antiviral activity of Dcr2. However, this could also be due to the differences in the experimental setup of RVFV versus BUNV.

Based on the strong differences in RVFV infection in KN- and GR-transfected cells, we hypothesized that the KN mutant is still able to produce 21 nt vsiRNAs after RVFV infection. However, small RNA analysis of KN expressing cells infected with RVFV showed no 21 nt vsiRNA peak for any segment (Fig. 2). In contrast, the positive control expressing WT Dcr2 resulted in 21 nt vsiRNA production for all three segments, with the highest amount mapping to the M segment, which is consistent with the previous observation of RVFV-infected Aag2 cells [30] and in line with the observed antiviral activity of WT Dcr2 against RVFV. The K39N (KN) mutation in *Ae. aegypti* Dcr2 is the homologous mutation of K34N in *Drosophila melanogaster* Dcr2 and is located in the helicase domain. More specifically, it has been shown that this aa is essential for the ATP binding in *D. melanogaster* Dcr2 and the K34N mutant is known as ATPase defective. The main function of the Dcr2 helicase domain is binding of blunt-end dsRNA, which is ATP dependent. In the absence of ATP or in case of the ATPase-defective mutant, blunt-end dsRNA cannot efficiently be bound, but instead, 3′ end overhang dsRNA is bound and cut into 22 nt instead of 21 nt siRNAs [23,24]. Interestingly, a peak of 22 nt RVFV-specific small RNAs for the S segment was detected in cells expressing the KN mutant. However, the same peak of 22 nt vRNAs was also present in the other helicase mutant, GR, as well as the negative control lacking Dcr2 expression at all. Even cells expressing WT Dcr2 showed the production of RVFV-specific 22 nt vRNAs for the S segment, although the amount was similar to the 21 nt vsiRNA peak. Such production of 22 nt vRNAs for the helicase mutants or the negative control lacking Dcr2 has not been observed for SFV-infected cells [28], supporting an RVFV-specific observation (independent of Dcr2), although other viruses would need to be tested for a final conclusion.

The two ORFs of the RVFV S segment are separated by a stem-loop structure, which was previously shown to be a main producer of 21 nt vsiRNAs [30]. Similar targeting of the stem-loop structure (especially from the genome) and production of 21 vsiRNAs have been observed in *Ae. aegypti* Dcr2 KO cells, independent of expressed Dcr2 proteins (none, WT or helicase mutants) (Fig. 3). As opposed to the exo-siRNA pathway, which relies on vRNA as the source dsRNA, the endogenous (endo-)siRNA pathway uses either sense-antisense dsRNA formed by transposon sequences or stem-loop RNA transcripts [54]. The miRNA pathway utilizes primary (pri-)miRNAs, which are mostly transcribed as ssRNAs possessing hairpin structures and processed into precursor (pre-)miRNA duplexes before being taken up and cut by Dicer-1 (Dcr1) into 22–23 nt mature miRNAs [55,56]. In both the endo-siRNA pathway and the miRNA pathway, hairpin RNA is efficiently recognized and cleaved by either Dcr2 or Dcr1, resulting in the production of 21 nt siRNAs and 22–23 nt miRNAs, respectively [54,56]. Therefore, the presence of 22 nt RVFV-specific RNAs in all treatments regardless of the presence of Dcr2 might suggest the involvement of Dcr1. It is unknown whether these 22 nt vRNAs are biologically functional, nor why the KN mutant retains antiviral activity in contrast to GR despite showing no significant differences in vRNA production. Further studies are needed to understand this in detail.

Ping-pong-produced sense and antisense vpiRNAs typically exhibit a 10 nt overlap with a U_1_ bias in the antisense and an A_10_ bias in the sense strand [15,28, 50, 57]. A notable increase in vRNA production of 25–30 nt length, with ping-pong-produced vpiRNA characteristics (Fig. 4), predominantly for the M segment, is observed in the presence of functional Dcr2, suggesting that Dcr2 and/or the vsiRNAs produced by Dcr2 are somehow linked to vpiRNA production. However, more research is needed to fully understand the extent of this involvement.

In summary, these results support the previous observations reported for the positive-strand RNA virus, SFV, and extend the importance of the different domains in Dcr2 to both positive- and negative-strand RNA viruses of the *Bunyaviricetes*. Furthermore, it highlights important characteristics in the interaction of the antiviral exo-siRNA pathway and RVFV. Nonetheless, more research is needed to understand the exo-siRNA pathway and the specific functions of the Dcr2 domains in detail. This study focuses on the effects of mutant Dcr2 on RVFV and BUNV in mosquito-derived cells. Although previous research has shown that results observed in *Ae. aegypti*-derived cells are the same or at least similar in mosquitoes, further research is needed to determine this for the Dcr2 mutants.

## supplementary material

10.1099/jgv.0.002046 Uncited Supplementary Material 1.

## Abbreviations

BUNV: Bunyamwera orthobunyavirus
CHIKV: chikungunya virus
DENV: dengue virus
exo-siRNA: exogenous small interfering RNA
hpi: h post-infection
hpt: h post-transfection
KO: knock-out
miRNAs: micro RNAs
PAZ: PIWI–Argonaute–Zwille
piRNAs: PIWI-interacting RNA
RNAi: RNA interference
RVFV: Rift Valley fever virus
SFV: Semliki Forest alphavirus
siRNA: small interfering RNA
vpiRNA: viral piRNA
vRNAs: viral RNAs
vsiRNAs: viral small interfering RNAs

## References

[1] Brady OJ, Hay SI (2020). The global expansion of dengue: how *Aedes aegypti* mosquitoes enabled the first pandemic arbovirus. Annu Rev Entomol.

[2] Carvalho FD, Moreira LA (2017). Why is *Aedes aegypti* Linnaeus so successful as a species?. Neotrop Entomol.

[3] Halstead SB (2019). Travelling arboviruses: a historical perspective. Travel Med Infect Dis.

[4] Weaver SC, Charlier C, Vasilakis N, Lecuit M (2018). Zika, chikungunya, and other emerging vector-borne viral diseases. Annu Rev Med.

[5] Weaver SC, Reisen WK (2010). Present and future arboviral threats. Antiviral Res.

[6] Pierson TC, Diamond MS (2020). The continued threat of emerging flaviviruses. Nat Microbiol.

[7] Huang Y-JS, Higgs S, Vanlandingham DL (2019). Emergence and re-emergence of mosquito-borne arboviruses. Curr Opin Virol.

[8] Zaid A, Burt FJ, Liu X, Poo YS, Zandi K (2021). Arthritogenic alphaviruses: epidemiological and clinical perspective on emerging arboviruses. Lancet Infect Dis.

[9] Kemp C, Imler J-L (2009). Antiviral immunity in drosophila. Curr Opin Immunol.

[10] Aliyari R, Ding S-W (2009). RNA-based viral immunity initiated by the Dicer family of host immune receptors. Immunol Rev.

[11] Elbashir SM, Harborth J, Lendeckel W, Yalcin A, Weber K (2001). Duplexes of 21-nucleotide RNAs mediate RNA interference in cultured mammalian cells. Nature.

[12] Okamura K, Ishizuka A, Siomi H, Siomi MC (2004). Distinct roles for Argonaute proteins in small RNA-directed RNA cleavage pathways. Genes Dev.

[13] Merkling SH, Crist AB, Henrion-Lacritick A, Frangeul L, Gausson V (2022). Multifaceted contributions of Dicer2 to arbovirus transmission by *Aedes aegypti*. bioRxiv.

[14] Samuel GH, Pohlenz T, Dong Y, Coskun N, Adelman ZN (2023). RNA interference is essential to modulating the pathogenesis of mosquito-borne viruses in the yellow fever mosquito *Aedes aegypti*. Proc Natl Acad Sci U S A.

[15] Varjak M, Maringer K, Watson M, Sreenu VB, Fredericks AC (2017). *Aedes aegypti* Piwi4 is a noncanonical PIWI protein involved in antiviral responses. mSphere.

[16] Leggewie M, Scherer C, Altinli M, Gestuveo RJ, Sreenu VB (2023). The *Aedes aegypti* RNA interference response against Zika virus in the context of co-infection with dengue and chikungunya viruses. PLoS Negl Trop Dis.

[17] Varjak M, Donald CL, Mottram TJ, Sreenu VB, Merits A (2017). Characterization of the Zika virus induced small RNA response in *Aedes aegypti* cells. PLoS Negl Trop Dis.

[18] Scherer C, Knowles J, Sreenu VB, Fredericks AC, Fuss J (2021). An *Aedes aegypti*-derived Ago2 knockout cell line to investigate arbovirus infections. Viruses.

[19] Mueller S, Gausson V, Vodovar N, Deddouche S, Troxler L (2010). RNAi-mediated immunity provides strong protection against the negative-strand RNA vesicular stomatitis virus in *Drosophila*. Proc Natl Acad Sci U S A.

[20] Weber F, Wagner V, Rasmussen SB, Hartmann R, Paludan SR (2006). Double-stranded RNA is produced by positive-strand RNA viruses and DNA viruses but not in detectable amounts by negative-strand RNA viruses. J Virol.

[21] Kidwell MA, Chan JM, Doudna JA (2014). Evolutionarily conserved roles of the dicer helicase domain in regulating RNA interference processing. J Biol Chem.

[22] Shabalina SA, Koonin EV (2008). Origins and evolution of eukaryotic RNA interference. Trends Ecol Evol.

[23] Sinha NK, Trettin KD, Aruscavage PJ, Bass BL (2015). Drosophila dicer-2 cleavage is mediated by helicase- and dsRNA termini-dependent states that are modulated by Loquacious-PD. Mol Cell.

[24] Sinha NK, Iwasa J, Shen PS, Bass BL (2018). Dicer uses distinct modules for recognizing dsRNA termini. Science.

[25] Ma J-B, Ye K, Patel DJ (2004). Structural basis for overhang-specific small interfering RNA recognition by the PAZ domain. Nature.

[26] Ji X (2008). The mechanism of RNase III action: how dicer dices. Curr Top Microbiol Immunol.

[27] Sasaki T, Shimizu N (2007). Evolutionary conservation of a unique amino acid sequence in human DICER protein essential for binding to Argonaute family proteins. Gene.

[28] Gestuveo RJ, Parry R, Dickson LB, Lequime S, Sreenu VB (2022). Mutational analysis of *Aedes aegypti* Dicer 2 provides insights into the biogenesis of antiviral exogenous small interfering RNAs. PLoS Pathog.

[29] Reuter M, Parry RH, McFarlane M, Gestuveo RJ, Arif R (2024). The PAZ domain of *Aedes aegypti* Dicer 2 is critical for accurate and high-fidelity size determination of virus-derived small interfering RNAs. Microbiology.

[30] Dietrich I, Jansen S, Fall G, Lorenzen S, Rudolf M (2017). RNA Interference restricts rift valley fever virus in multiple insect systems. *mSphere*.

[31] Dietrich I, Shi X, McFarlane M, Watson M, Blomström A-L (2017). The antiviral RNAi response in vector and non-vector cells against orthobunyaviruses. PLoS Negl Trop Dis.

[32] Altinli M, Leggewie M, Schulze J, Gyanwali R, Badusche M (2023). Antiviral RNAi response in *Culex quinquefasciatus*-derived HSU cells. Viruses.

[33] Gerrard SR, Nichol ST (2007). Synthesis, proteolytic processing and complex formation of N-terminally nested precursor proteins of the Rift Valley fever virus glycoproteins. Virology.

[34] Mottram TJ, Li P, Dietrich I, Shi X, Brennan B (2017). Mutational analysis of Rift Valley fever phlebovirus nucleocapsid protein indicates novel conserved, functional amino acids. PLoS Negl Trop Dis.

[35] Kading RC, Crabtree MB, Bird BH, Nichol ST, Erickson BR (2014). Deletion of the NSm virulence gene of Rift Valley fever virus inhibits virus replication in and dissemination from the midgut of *Aedes aegypti* mosquitoes. PLoS Negl Trop Dis.

[36] Billecocq A, Spiegel M, Vialat P, Kohl A, Weber F (2004). NSs protein of Rift Valley fever virus blocks interferon production by inhibiting host gene transcription. J Virol.

[37] Terasaki K, Ramirez SI, Makino S (2016). Mechanistic insight into the host transcription inhibition function of Rift Valley fever virus NSs and its importance in virulence. PLoS Negl Trop Dis.

[38] Lerolle S, Freitas N, Cosset F-L, Legros V (2021). Host cell restriction factors of bunyaviruses and viral countermeasures. Viruses.

[39] Ly HJ, Ikegami T (2016). Rift Valley fever virus NSs protein functions and the similarity to other bunyavirus NSs proteins. Virol J.

[40] Balkema-Buschmann A, Rissmann M, Kley N, Ulrich R, Eiden M (2018). Productive propagation of Rift Valley fever phlebovirus vaccine strain MP-12 in Rousettus aegyptiacus fruit bats. Viruses.

[41] Tercero B, Terasaki K, Narayanan K, Makino S (2023). Mechanistic insight into the efficient packaging of antigenomic S RNA into Rift Valley fever virus particles. Front Cell Infect Microbiol.

[42] Szemiel AM, Failloux A-B, Elliott RM (2012). Role of bunyamwera orthobunyavirus NSs protein in infection of mosquito cells. PLoS Negl Trop Dis.

[43] Charlton FW, Hover S, Fuller J, Hewson R, Fontana J (2019). Cellular cholesterol abundance regulates potassium accumulation within endosomes and is an important determinant in bunyavirus entry. J Biol Chem.

[44] Shi X, Elliott RM (2009). Generation and analysis of recombinant Bunyamwera orthobunyaviruses expressing V5 epitope-tagged L proteins. J Gen Virol.

[45] Barr JN, Elliott RM, Dunn EF, Wertz GW (2003). Segment-specific terminal sequences of Bunyamwera bunyavirus regulate genome replication. Virology.

[46] Fredericks AC, Russell TA, Wallace LE, Davidson AD, Fernandez-Sesma A (2019). *Aedes aegypti* (Aag2)-derived clonal mosquito cell lines reveal the effects of pre-existing persistent infection with the insect-specific bunyavirus Phasi Charoen-like virus on arbovirus replication. PLoS Negl Trop Dis.

[47] Varela M, Schnettler E, Caporale M, Murgia C, Barry G (2013). Schmallenberg virus pathogenesis, tropism and interaction with the innate immune system of the host. PLoS Pathog.

[48] Chen S, Zhou Y, Chen Y, Gu J (2018). fastp: an ultra-fast all-in-one FASTQ preprocessor. Bioinformatics.

[49] Langmead B, Salzberg SL (2012). Fast gapped-read alignment with Bowtie 2. Nat Methods.

[50] Antoniewski C (2014). Computing siRNA and piRNA overlap signatures. Methods Mol Biol.

[51] Crooks GE, Hon G, Chandonia J-M, Brenner SE (2004). WebLogo: a sequence logo generator. Genome Res.

[52] Quinlan AR, Hall IM (2010). BEDTools: a flexible suite of utilities for comparing genomic features. Bioinformatics.

[53] Turell MJ, Rossi CA (1991). Potential for mosquito transmission of attenuated strains of Rift Valley fever virus. Am J Trop Med Hyg.

[54] Donald CL, Kohl A, Schnettler E (2012). New insights into control of arbovirus replication and spread by insect RNA interference pathways. Insects.

[55] Breving K, Esquela-Kerscher A (2010). The complexities of microRNA regulation: mirandering around the rules. Int J Biochem Cell Biol.

[56] O’Brien J, Hayder H, Zayed Y, Peng C (2018). Overview of MicroRNA biogenesis, mechanisms of actions, and circulation. Front Endocrinol.

[57] Varjak M, Leggewie M, Schnettler E (2018). The antiviral piRNA response in mosquitoes?. J Gen Virol.

