# Supplementary material for: Dicer-2 mutations in Aedes aegypti cells lead to a diminished antiviral function against Rift Valley fever virus and Bunyamwera virus infection
Source: J Gen Virol. 2024 Nov 7;105(11):002046. doi: 10.1099/jgv.0.002046 (PMC11542633; doi:10.1099/jgv.0.002046)
Supplement: Uncited Supplementary Material 1. [file jgv-105-02046-s001.pdf]

Supplementary data Dornbusch, Reuters et al.:

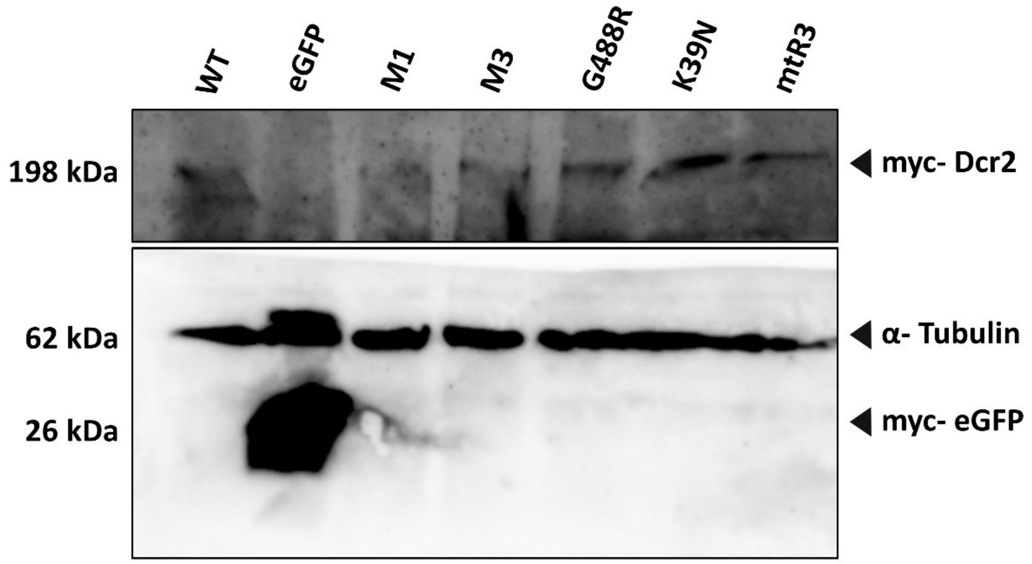

Fig. S1. Western Blot for assessment of Dcr2 expression after WT or mutant Dcr2 transfection in Dcr2 KO cell line

Method: SDS-PAGE and Western Blotting (Tank Blot)

Aag2-AF319 cells were seeded in duplicate at 2 x 10^5 cells/well in 24-well plates and allowed to settle. The transfection was performed with 500 ng plasmid expressing WT or mutant Dcr2 (with pPUb-myc-eGFP as control) and 1 µL DharmaFECT 2 (Horizon Discovery). Cell lysis was performed at 24 hpi using 4x Laemmli sample buffer (BioRad) according to the manufacturers protocol. The cell lysate was separated using a 4-15 % Mini-PROTEAN® TGX™ Precast Protein Gel (BioRad) for 30 min at 200 V. For transferring proteins onto a nitrocellulose membrane, a Mini-Trans® Blot Cell (BioRad) was used for Tank Blot application at 400 mA for 2 h. The membranes were blocked for at least 1 h using 2% (w/v) non-fat dry milk powder in Tween-PBS (PBS with 0.1% Tween 20) and then washed three times for 5 min each using 0.1% (v/v) PBS-Tween20 (PBS-T). Primary antibodies were from mouse, anti-myc tag antibody (1:1000) and mouse anti-α tubulin antibody (1:10000). Antibodies were diluted in 0.2% (w/v) non-fat dry milk powder and Tween-PBS. After 1 h, the membranes were again washed three times in PBS-T for 5 min, each. This was followed by another incubation of 1 h with goat anti-mouse secondary antibody (1:10000) conjugated with horseradish peroxidase (HRP) in 0.2% (w/v) non-fat dry milk powder in Tween-PBS. The membranes were again washed three times

with PBS-T and developed using the SuperSignal® West Pico Chemieluminescent Substrate (Thermo Fisher Scientific) according to the manufacturer´s protocol and the ChemieDoc MP Imaging System (BioRad).

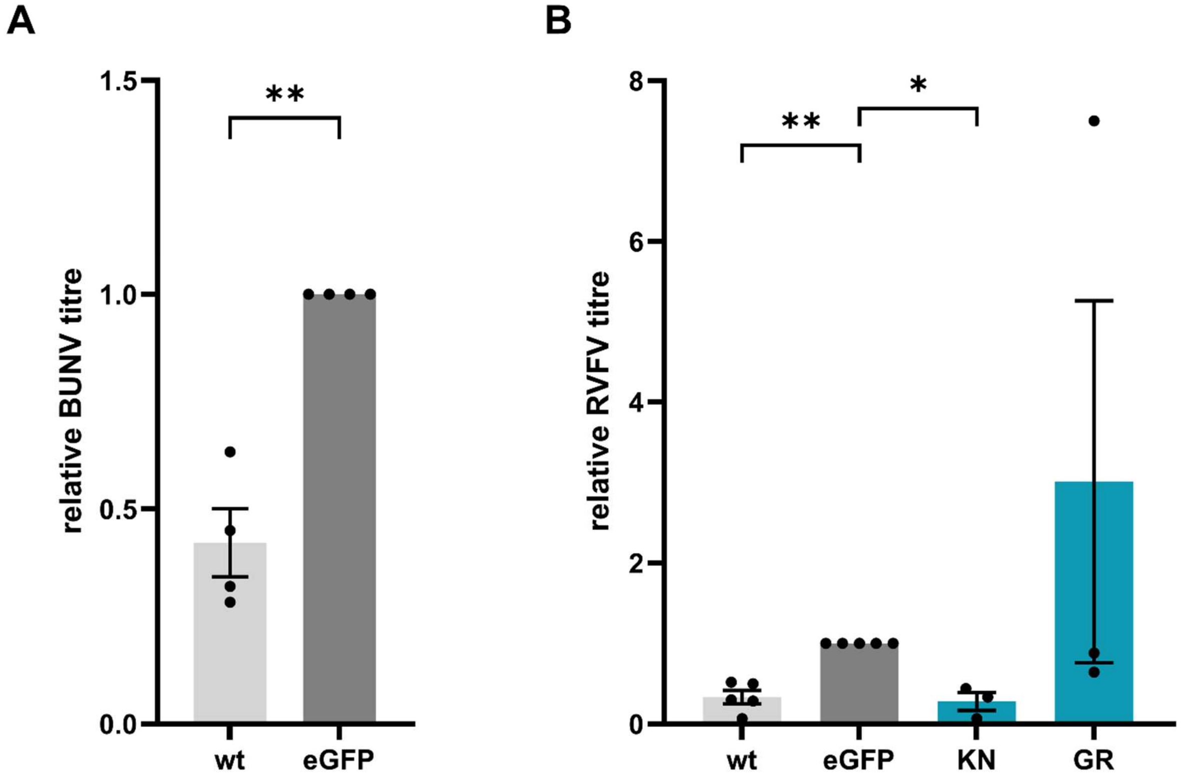

Fig. S2. Effect of Dcr2 mutants or Dcr2 wt on BUNV and RVFV production in Aag2-AF319 cells. (A) Effect of WT Dcr2 on BUNV and **(B)** of WT and mutant Dcr2 on RVFV with pPUb-myc-eGFP as negative control. Aag2-AF319 cells were transfected with pPUb-plasmids expressing WT or mutant Dcr2s, with pPUb-myc-eGFP as control. Cells were infected at 24 hpt with BUNV or RVFV (both MOI 0.1), respectively. At 72 hpi, supernatant was collected and infectious virus particles determined by plaque assays in BHK-21 cells. Plaque forming units (PFU)/mL were calculated and subsequently normalized to the eGFP control. Results are shown as relative BUNV or RVFV titres at 72 hpi ± SEM of at least 3 independent biological replicates. The dots represent different biological replicates, consisting of two technical replicates each with * = $p<0.05$, ** = $p<0.01$ as according to Student's t test.

Method: Plaque assay using Avicel

Supernatant of WT or mutant Dcr2 transfected Aag2-AF319 cells infected with either BUNV or RVFV was collected at 72 hpi prior to the qPCR experiments (Fig. 1). For the plaque assays, 2 x 10^5 BHK-21 cells were seeded per well in a 12-well plate. After 24 h, medium was removed and serial dilutions of BUNV or RVFV supernatant were added. At 1 hpi, 2 mL of a previously prepared 1:1 mix of 1.2% Avicel (FMC International, Little Island, Ireland) and 4% medium, made up of 2X MEM (Gibco ThermoFisher Scientific Inc., Waltham, MA, USA) and 4% fetal

bovine serum (FBS; Gibco Capricorn Scientific GmbH, Ebsdorfergrund, Germany), was added to each well. The plates were allowed to incubate for 96 h at 37°C with 5% CO_2, at which point both RVFV and BUNV are expected to form easily visible plaques. Plates were fixed using 8% formaldehyde for 1 h, after which they were stained using crystal violet for another hour. Plaques were counted, plaque forming units (PFU) per mL were calculated and normalized to the eGFP negative control. The results were plotted and statistically tested using Student's t test via GraphPad Prism 10 (GraphPad Software, Boston, MA, USA).

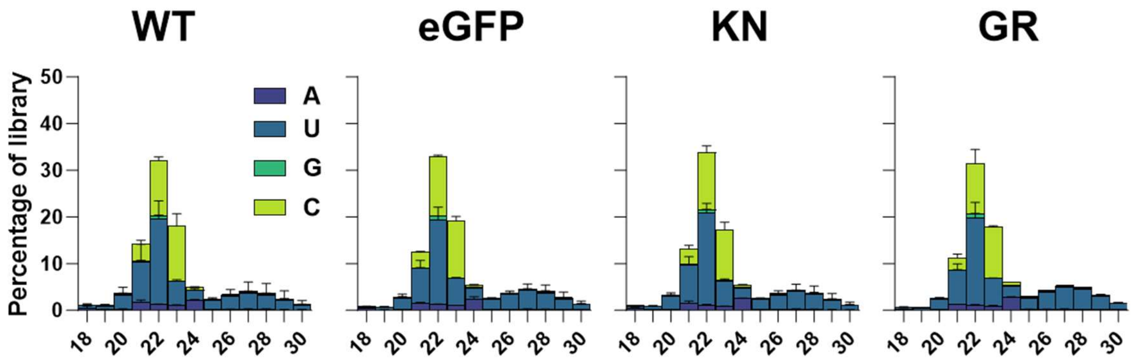

Fig. S3. Size distribution of the raw smallRNA sequencing data libraries and first position base bias. Each treatment is the average of two independent sequencing runs (n=2). Error bars indicate +/- SD.

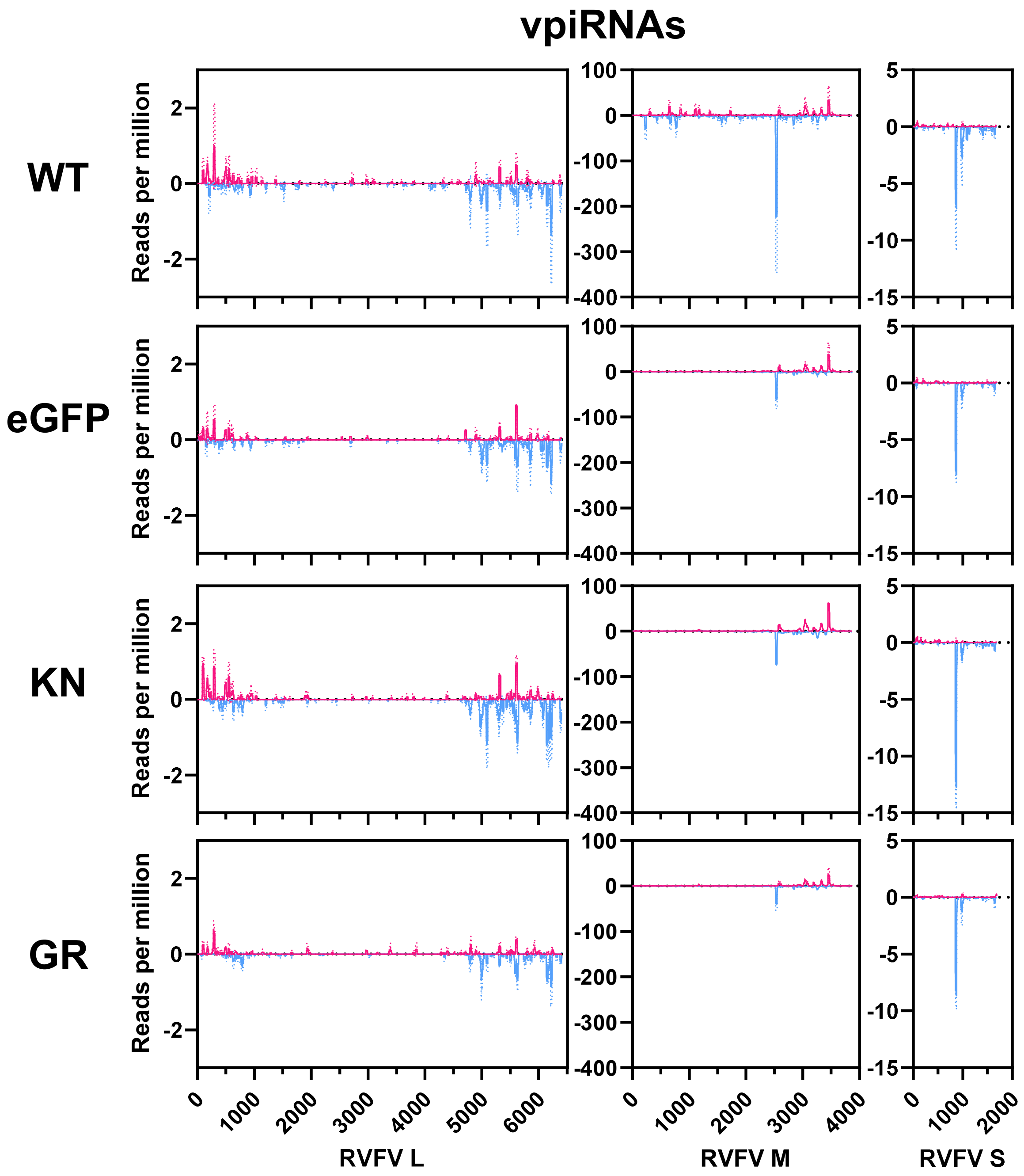

Fig. S4. Differences in the mapping of vpiRNAs in WT Dcr2, KN and GR mutants and Dcr2 negative eGFP in RVFV infection. Coverage of RVFV-derived 25-30 nt vpiRNAs over the antigenome (magenta) or genome (cyan) (Y axis, vpiRNA reads per million) for the L, M and S segments of RVFV from two independent experiments +/- SD.

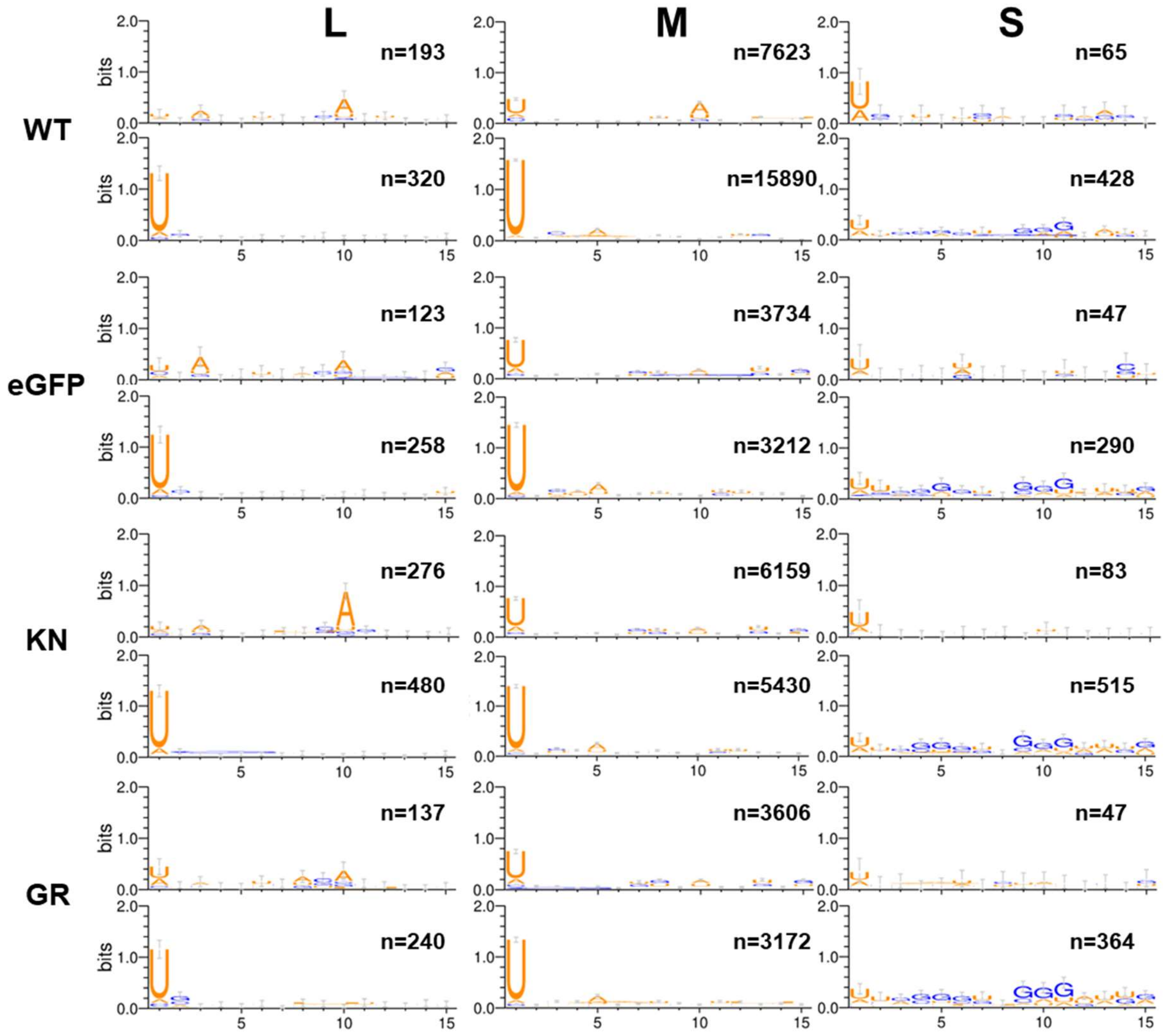

Fig. S5. RVFV-specific piRNA small RNAs in WT and mutant of Aag2-AF319 cells transiently expressing WT Dcr2, KN and GR mutations and eGFP control infected with RVFV. Representation of sequence logos for each segment with 25-30nt reads trimmed to the first 15nt. All reads matching the RVFV MP12 antigenomic (top) and genomic (bottom) sequences were analyzed for their base frequencies at each position. Overall height of the stack indicates conservation presented as bits, with the height of each symbol reflecting the relative frequency of the corresponding nucleotide at a given position. Error bars indicate an approximate 95% credible interval. Number of reads across two replicates are given on the figure.

S1 Table. Primer sequence.

Target	Primer name	Sequence (5‘ – 3‘)
RVFV	FW	TGAAAATTCCTGAGACACATGG
	RV	ACTTCCTTGCATCATCTGATG
	Probe e-FAM	CAATGTAAGGGGCCTGTGTGGACTTGTG
S7 (RVFV)	FW	CCAGGCTATCCTGGAGTTG
	RV	CCAGGCTATCCTGGAGTTG
	Probe e-HEX	HEX-TCGTGAGATCGAGTTCAACAGCAAGAAGGC -BHQ1
BUNV	FW	CCCACACACAGTCAGTAACAACA
	RV	CCCACACACAGTCAGTAACAACA
S7 (BUNV)	FW	CCAGGCTATCCTGGAGTTG
	RV	GACGTGCTTGCCGGAGAAC

S2 Table. Sequencing and library statistics for small RNA (size range 18-30 nt) were used in this study and SRA accession. The number of mapped reads per segment and the percentage per replicate are given.

**Sample**	**SRA Accession**	**Reads**	**L (%)**	**M (%)**	**S (%)**	**Total (%)**
WT_1	SRR29288252	14412964	1080<br>0.01	16320<br>0.11	2086<br>0.01	0.14
WT_2	SRR29288251	12291491	728<br>0.01	22304<br>0.18	1165<br>0.01	0.20
eGFP_1	SRR29288250	11109292	606<br>0.01	6454<br>0.06	979<br>0.01	0.07
eGFP_2	SRR29288249	11949197	693<br>0.01	5704<br>0.05	1406<br>0.01	0.07
KN_1	SRR29288248	16024765	1017<br>0.01	10690<br>0.07	1559<br>0.01	0.08
KN_2	SRR29288247	11943823	701<br>0.01	7053<br>0.06	1657<br>0.01	0.08
GR_1	SRR29288246	18069573	1171<br>0.01	8105<br>0.04	2444<br>0.01	0.06
GR_2	SRR29288245	12274390	600<br>0.00	5766<br>0.05	1196<br>0.01	0.06

S3 Table. P-values of the analysis of antiviral activity of mutant Dcr2 against RVFV and BUNV.

BUNV							
	wt	eGFP	M1	M3	KN	GR	mtR3
wt	X	0.0365	0.4516	0.1991	0.1393	0.3059	0.1767
eGFP	0.0365	X	0.9745	0.6966	0.4139	0.8586	0.5540
M1	0.4516	0.9745	X	0.7841	0.5368	0.8821	0.6645
M3	0.1991	0.6966	0.7841	X	0.6719	0.9009	0.8400
KN	0.1393	0.4139	0.5368	0.6719	X	0.6140	0.8267
GR	0.3059	0.8586	0.8821	0.9009	0.6140	X	0.7620
mtR3	0.1767	0.5540	0.6645	0.8400	0.8267	0.7620	X
**RVFV**							
	wt	eGFP	M1	M3	KN	GR	mtR3
wt	X	0.0051	0.0171	0.1195	0.7307	0.1967	0.1108
eGFP	0.0051	X	0.1141	0.2989	0.0066	0.9494	0.5223
M1	0.0171	0.1141	X	0.6831	0.0176	0.3362	0.5982
M3	0.1195	0.2989	0.6831	X	0.1239	0.3352	0.4941
KN	0.7307	0.0066	0.0176	0.1239	X	0.2102	0.1171
GR	0.1967	0.9494	0.3362	0.3352	0.2102	X	0.6588
mtR3	0.1108	0.5223	0.5982	0.4941	0.1171	0.6588	X
